# Fabrication of slender elastic shells by the coating of curved surfaces

**DOI:** 10.1038/ncomms11155

**Published:** 2016-04-04

**Authors:** A. Lee, P. -T. Brun, J. Marthelot, G. Balestra, F. Gallaire, P. M. Reis

**Affiliations:** 1 Department of Mechanical Engineering, Massachusetts Institute of Technology, Cambridge, Massachusetts 02139, USA; 2 Department of Mathematics, Massachusetts Institute of Technology, Cambridge, Massachusetts 02139, USA; 3 Department of Civil and Environmental Engineering, Massachusetts Institute of Technology, Cambridge, Massachusetts 02139, USA; 4 Laboratory of Fluid Mechanics and Instabilities, EPFL, CH1015 Lausanne, Switzerland

## Abstract

Various manufacturing techniques exist to produce double-curvature shells, including injection, rotational and blow molding, as well as dip coating. However, these industrial processes are typically geared for mass production and are not directly applicable to laboratory research settings, where adaptable, inexpensive and predictable prototyping tools are desirable. Here, we study the rapid fabrication of hemispherical elastic shells by coating a curved surface with a polymer solution that yields a nearly uniform shell, upon polymerization of the resulting thin film. We experimentally characterize how the curing of the polymer affects its drainage dynamics and eventually selects the shell thickness. The coating process is then rationalized through a theoretical analysis that predicts the final thickness, in quantitative agreement with experiments and numerical simulations of the lubrication flow field. This robust fabrication framework should be invaluable for future studies on the mechanics of thin elastic shells and their intrinsic geometric nonlinearities.

## Abstract (web summary)

Foods industry calls for effective methods to prepare thin shell structures, such as hollow chocolate eggs, which are also relevant to many industrial applications. Here, Lee *et al*. show how to fabricate hemispherical shells with nearly uniform yet tunable thickness to produce elastic structures.

## Graphical abstract

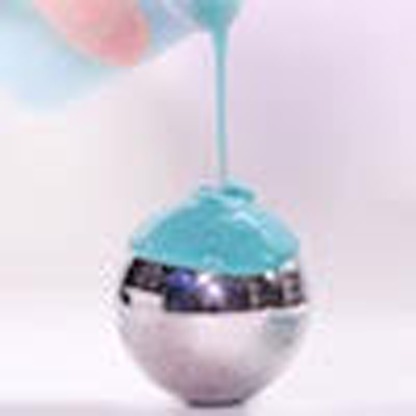

Hollow chocolate eggs, rabbits and bonbons have been fabricated since the 1600s by pouring molten chocolate into a mold and draining the excess. Solidification upon cooling ceases the flow and results in a solid shell of nearly constant thickness^1^. Beyond chocolatiers, the polymer industry abounds with needs to fabricate thin shell structures, and a plethora of manufacturing processes have been developed for this purpose, including: injection^2^, rotational^1^ and blow molding^3^, as well as dip coating^4^. Common to all of the above techniques are limitations in the thickness of the shells (e.g., ∼0.5 mm for injection molding) and its uniformity (typically ∼20% for rotational molding^5^), as well as a striking lack of predictive theoretical models due to the multi-physics complexity of the processes. Rotational molding, for example, involves coating the inner surface of a hollow mold with a polymer melt, which is then rotated biaxially while applying a decreasing heating profile until a solid shell is formed^1^. As another example, injection molding, is geared for mass-production manufacturing and requires costly precision-machined molds that are inflexible to variations in the geometry of the part^2^. In these processes, the optimization of the control parameters is largely tuned empirically, with compromises on versatility, predictability and reproducibility^5^. As such, these techniques are not directly applicable to laboratory settings, where adaptable, inexpensive and predictable rapid-prototyping tools are more desirable. This is particularly the case for the fabrication of thin, smooth and flexible three dimensional structures.

For flat and cylindrical surfaces, a variety of thin film coating techniques are well established^6^. A significant advantage for these geometries is that, when compared to their double-curved counterparts, they are more amenable to theoretical modeling to predict how the final film thickness depends on the control parameters^7^,^8^. In these cases, the flow driven by viscous stresses and held by capillary forces is ‘frozen' as the media cools, cures or dries, yielding a defect-free and uniform finish. As a result, these robust coating techniques have matured to be ubiquitous in industry. To generate (ultra-) thin sheets, spin-coating is now widespread (e.g., in microfluidics) to attain constant and tunable film thicknesses^9^,^10^. Similarly, spin-casting exploits centrifugal forces on a rotating cylindrical surface to evenly distribute a polymer solution and fabricate nearly constant thickness shells in a highly controllable manner^11^. This technique was instrumental in identifying the role of imperfections on the critical buckling conditions of cylindrical shells in the 1960s (refs 12, 13). For double-curved surfaces, there is a need for simple and versatile fabrication methods that are analogous to the coating of fibers, plates and cylinders and able to yield uniform, controllable and predictable results.

Here, we introduce a simple and robust mechanism to fabricate hemispherical thin elastic shells by the coating, drainage and subsequent curing of polymer solutions on curved molds. Our process is analogous to spin-coating (itself not applicable on curved surfaces), albeit with a gravity-driven flow in lieu of centrifugal forces. Through a systematic series of experiments using elastomers, we show that drainage can lead to coatings that are ‘frozen' in time as the polymer cures, thereby leading to a nearly uniform thin elastic shell. A theoretical analysis of the underlying lubrication flow during drainage, which includes the evolution of the rheological properties of the polymer as it cures, is able to accurately predict the final thickness of the shell as a function of the material properties of the polymer and the geometry of the substrate. Importantly, the final shell thickness is found to be independent of the initial conditions such as the height of pouring and the volume of poured fluid, as well as the initial thickness profile. Moreover, we find that the shell thickness can be tuned over one order of magnitude by changing the waiting time between the preparation of the polymer solution and the moment of pouring onto the mold. Our analysis demonstrates that the robustness and flexibility of this mechanism are inherent consequences of the loss of memory in the flow field. Our approach provides a fast, robust and predictable mechanism to fabricate thin shells with flexibility in their material and geometric properties by tuning the control parameters.

## Results

### Elastic shells of uniform thickness from viscous coating

**Figure 1:**
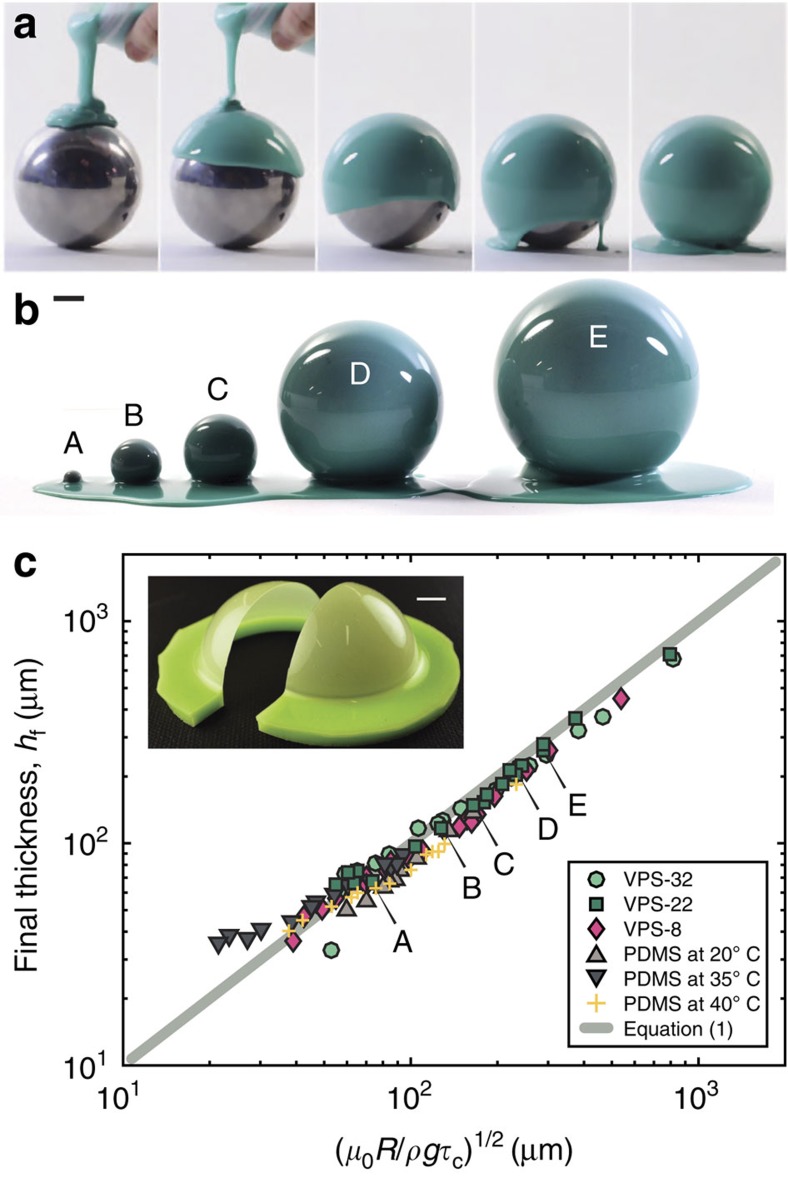
Coating process and resulting thickness of elastic shells. (**a**) Liquid VPS-22 (see Methods) is poured onto a sphere (*R*=38 mm), then drains under gravity and eventually cures to produce an elastic shell (see Supplementary Movie 1). The time interval between each frame is 2 s. (**b**) Similar procedure to that of (**a**) for spheres in a range of radii, 1≤*R*[mm]≤375. (**c**) Thickness of the elastic shells, *h*_f_, as a function of 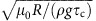, for various polymer solutions (VPS and PDMS) and temperatures (for PDMS). See Methods for details. The solid line corresponds to equation (1). Inset: An elastic shell is cut along a meridian for the thickness measurements. Scale bars, 10 mm.

In Fig. 1a, we present a series of photographs that illustrate our coating process. A silicone-based liquid polymer solution is poured onto a rigid sphere (mold), drains under the effect of gravity and eventually covers the surface. We used both vinylpolysiloxane (VPS) and polydimethylsiloxane (PDMS), at different mixing and curing conditions (see Methods for details), to achieve a variety of rheological properties. With time, cross-linking of the polymer film that emerges from the drainage process yields a thin elastic shell that can be readily peeled from the mold. The final thickness of these elastic shells *h*_f_ is found to be uniform (to within 6.6% (VPS) and 8.7% (PDMS) over the hemisphere).

The above procedure was repeated with molds in a range of radii (1≤*R*[mm]≤375, see Fig. 1b), and we found that *h*_f_∼*R*^1/2^, as shown in Fig. 1c. This result is robust and independent of either the details of the polymer or the curing temperature. The square-root dependence of *h*_f_ on *R* can be rationalized by balancing the characteristic curing time, 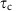, of the polymer solution and the characteristic drainage time, 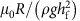, that is obtained when balancing the viscous stresses and gravity in the lubrication layer, such that


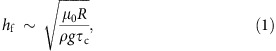


where *μ*_0_ is a characteristic viscosity of the polymer (e.g., its initial value), *ρ* its density, and *g* is the acceleration of gravity. The fact that all the data in Fig. 1c collapses onto a master curve (irrespective of the polymer and curing temperature, over a wide range of *R*) supports this scaling analysis. Below, we shall develop a theoretical description that more formally recovers this scaling, both analytically and numerically.

### The dynamics of coating

We proceed by experimentally characterizing the coating dynamics that, upon curing of the polymer, results in a thin elastic shell (Fig. 1a). As a representative case, we focus on VPS Elite 32 (hereafter referred to as VPS-32, see Methods) poured onto a sphere with *R*=38 mm. A broader exploration with other silicone-based polymers is provided in the Supplementary Table 1, as well as Supplementary Figs 1 and 5, nonetheless, yielding similar results.

**Figure 2:**
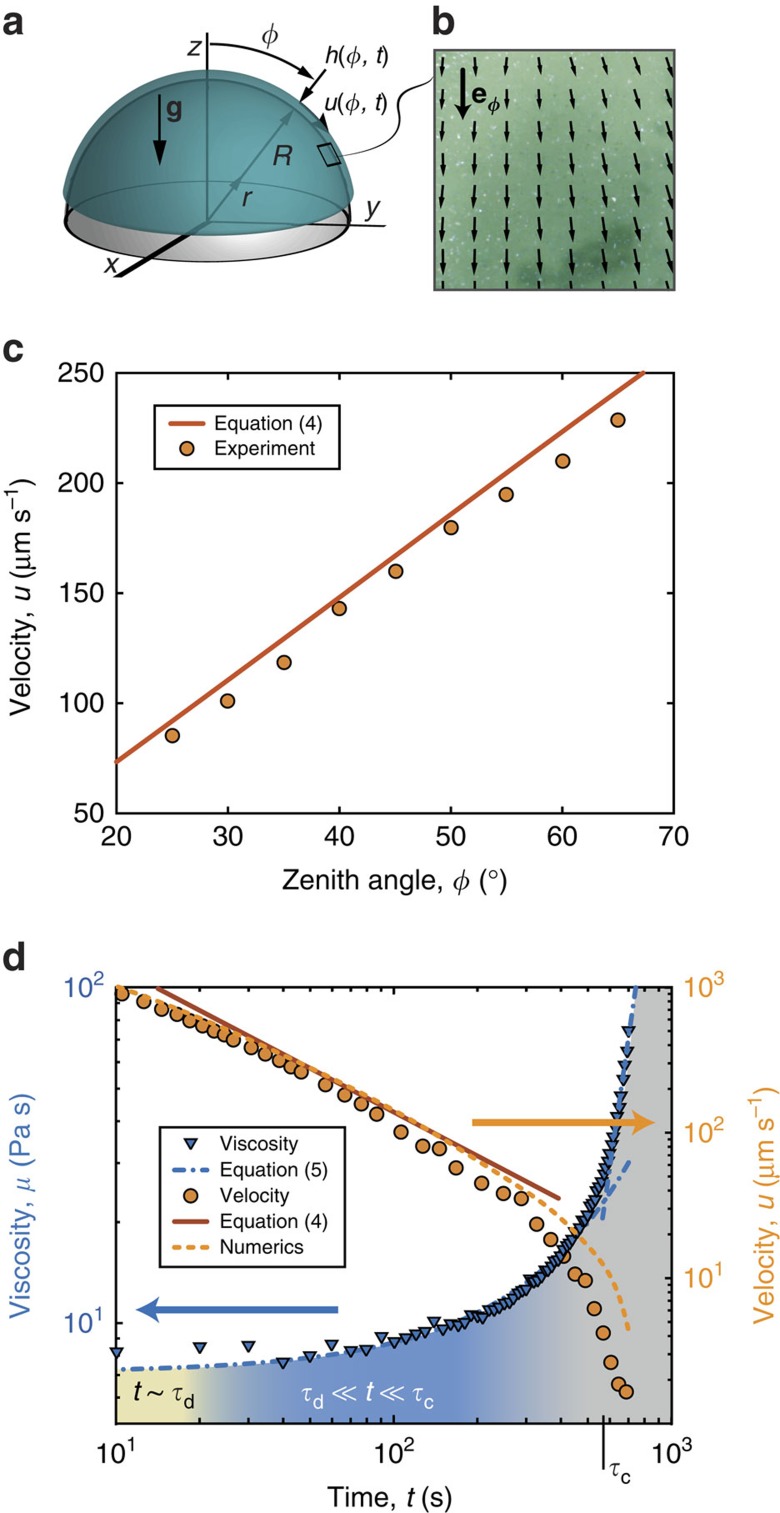
Spatial and temporal variation of the flow velocity. (**a**) Schematic diagram of the coating problem; *h*(*φ*, *t*) is the thickness of the viscous film and *u*(*φ*, *t*) is the flow velocity during drainage. (**b**–**d**) All data is for VPS-32 at 20 °C. (**b**) Instantaneous velocity field at *t*=60 s in a 1 × 1 cm^2^ region of the film located at *φ*=60° of a sphere (*R*=38 mm), obtained through PIV. (**c**) Dependence of the instantaneous local velocity (at *t*=60 s) on *φ*. (**d**) Time variation of the velocity, *u*(*φ*=60°, *t*) orange circles, and the viscosity, *μ*(*t*) blue triangles, of the polymer. The characteristic curing time, *τ*_c_, separates the drainage and curing regimes for both *u*(*φ*, *t*) and *μ*(*t*). The dash-dot line is the best fit for the viscosity: equation (5) with *μ*_0_=7.1±0.2 Pa s, *α*=5.3±0.7, *β*=(2.06±0.09) × 10^−3^, and 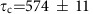 s. The solid and dashed lines are the predictions from our model for the velocity field using equation (4) and direct numerical simulations, respectively.

A schematic diagram of our system is presented in Fig. 2a, for a hemispherical mold, aligned such that gravity is parallel to the axis that connects its center to the pole; **g**=−*g* **e**_*z*_. Both the local thickness, *h*(*φ*, *t*), and the free surface velocity, *u*(*φ*, *t*), of the draining film are assumed axisymmetric and vary in both time, *t*, and space (i.e., zenith angle, *φ*). At the pole (*φ*=0), the fluid velocity vanishes due to symmetry. Elsewhere, the velocity is predominantly in the longitudinal direction, **e**_*φ*_. This is supported by the representative velocity field shown in Fig. 2b, obtained through PIV (see Methods), at *t*=60 s in a 1 × 1 cm^2^ region of the film located at *φ*=60°. Moreover, the instantaneous local velocity is found to increase with *φ* (Fig. 2c).

In Fig. 2d, we plot the time-series of the free surface velocity at the specific location *u*(*φ*=60°, *t*); the flow progressively slows down and eventually comes to a halt in finite time. This leaves a coating of the final thickness, *h*_f_, on the mold. The velocity profile and its arrest are found to correlate directly to the change in the viscosity, *μ*, as the polymer cures (Fig. 2d), which was determined through the rheometry at the appropriate shear rate (see Methods). Note that the initial drainage and subsequent curing regimes are separated by the characteristic curing time, 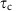, which is significantly larger than the initial drainage time, 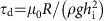, where *h*_i_ is the initial average coating thickness. For example, in the representative case above for VPS-32, we find 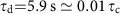 (using *h*_i_=2 mm, *R*=38 mm, *ρ*=1,160 kg m^−3^, *μ*_0_=7.1 Pa s and 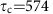 s). A direct consequence of this separation of timescales is that there is loss of memory in the process, such that *h*_f_ should be independent of *h*_i_. This prediction will be thoroughly examined in the *Discussion*, below. Returning to the time evolution of *u* and *μ* (Fig. 2d), at early times 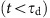 there are some disturbances due to initial conditions and we do not attempt to describe this regime. During intermediates times 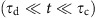, *μ* is approximately constant, and the velocity is set by viscous drainage with *u*∼1/*t* (ref. 14). For 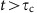, as the curing of the polymer accelerates, *μ* increases sharply with time, and consequently, the flow velocity slows down dramatically.

The separation of the drainage and curing timescales can be leveraged to further tune the final thickness of the shell. Since *h*_f_ is dictated by the interplay between the drainage timescale and polymerization timescale, 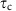, the final thickness can be increased by accelerating the curing process. One strategy to achieve this would be to alter 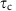 by modifying the kinetics of cross-linking (e.g., through additives or temperature), which would also modify the viscosity of the thin film or the elastic modulus of the final shell. An alternative is to shift the origin of the process by waiting for a time, 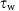, between the preparation of the polymer and the instant when the mixture is poured onto the mold. This waiting procedure offers an additional lever in tuning the properties of the fabricated shells.

Having presented our robust and versatile mechanism to fabricate thin elastic shells by the coating and subsequent curing of a polymer film, we proceed by rationalizing this process through a theoretical framework that is able to predict *h*_f_.

### Nonlinear drainage flow solution

It is well known that the thickness at the pole of a thin viscous film draining on a spherical surface is given by 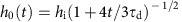 (refs 14, 15). We now seek to generalize this solution to account for the temporal and spatial variation (in *φ*) of the film. We shall first consider a Newtonian fluid and, once the nonlinear drainage flow solution is obtained, then include curing effects (i.e., the time dependence of *μ*(*t*) shown in Fig. 2d).

Starting with the lubrication equations^14^ that describe our axisymmetric flow on a hemispherical substrate and performing a nonlinear expansion of the form 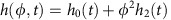 (see Supplementary Note 4) yields


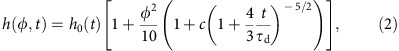


where *c* is a numerical factor that depends on the initial condition (e.g., *c*=−1 if the initial thickness profile is uniform). In the limit of 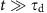, equation (2) simplifies to


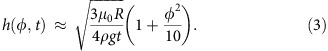


The memory loss of the flow mentioned earlier arising from the separation of the drainage and curing timescales is well captured by this description given that *h*_i_ is absent from equation (3). Moreover, there is a weak dependence on *φ* (8.7% s.d.); a general result that has also been observed in the thinning of an air bubble formed in a fluid bath^16^, as well as in the thin air layer that supports a drop bouncing on a fluid interface^17^.

As an indirect validation of equation (3), we substitute it into the free surface velocity equation describing the parabolic flow profile on a sphere, *u*=*ρgh*^2^sin*φ*/(2*μ*_0_)^14^, to obtain


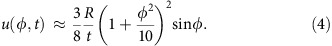


This prediction for the variation of *u* on both *φ* (at fixed *t*) and *t* (at fixed *φ*) is in agreement with the experimental velocity profiles shown in Fig. 2c,d for 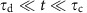 (i.e., in the regime after the initial drainage, when the polymer viscosity is approximately constant, and prior to curing). In particular, *u* is found to be almost linear in *φ* as the cubic term of the Taylor expansion is *φ*^3^/30 in lieu of the conventional *φ*^3^/6 of the sine. Strikingly, the velocity field in this regime is independent of both gravity and viscosity and is solely set by the geometry of the problem, so that no material parameters enter the prediction.

### Including the effects of curing into the flow solution

The curing of the polymer has not yet been taken into account in our model, which, as is, yields a vanishing coating thickness since equation (3) states that *h*∼1/*t*^1/2^. To do so, the above framework is modified by considering a time-varying viscosity^18^ using a piecewise function of the form


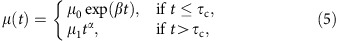


with 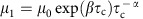 chosen to ensure continuity at 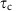 and where *β* and *α* are fitting parameters. Equation (5) is fitted to the experimental data and found to accurately describe the viscosity evolution (Fig. 2d). Combining this description for the viscosity with the lubrication equations yields a complete model for our system (the full details are provided in Supplementary Note 5), including an expression for the final thickness,


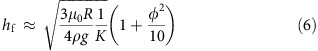


that is consistent with equation (3) but with 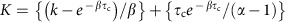 (instead of *K*=*t*), where *k*=1 when there is no delay between the preparation and the coating with the polymer solution.

**Figure 3:**
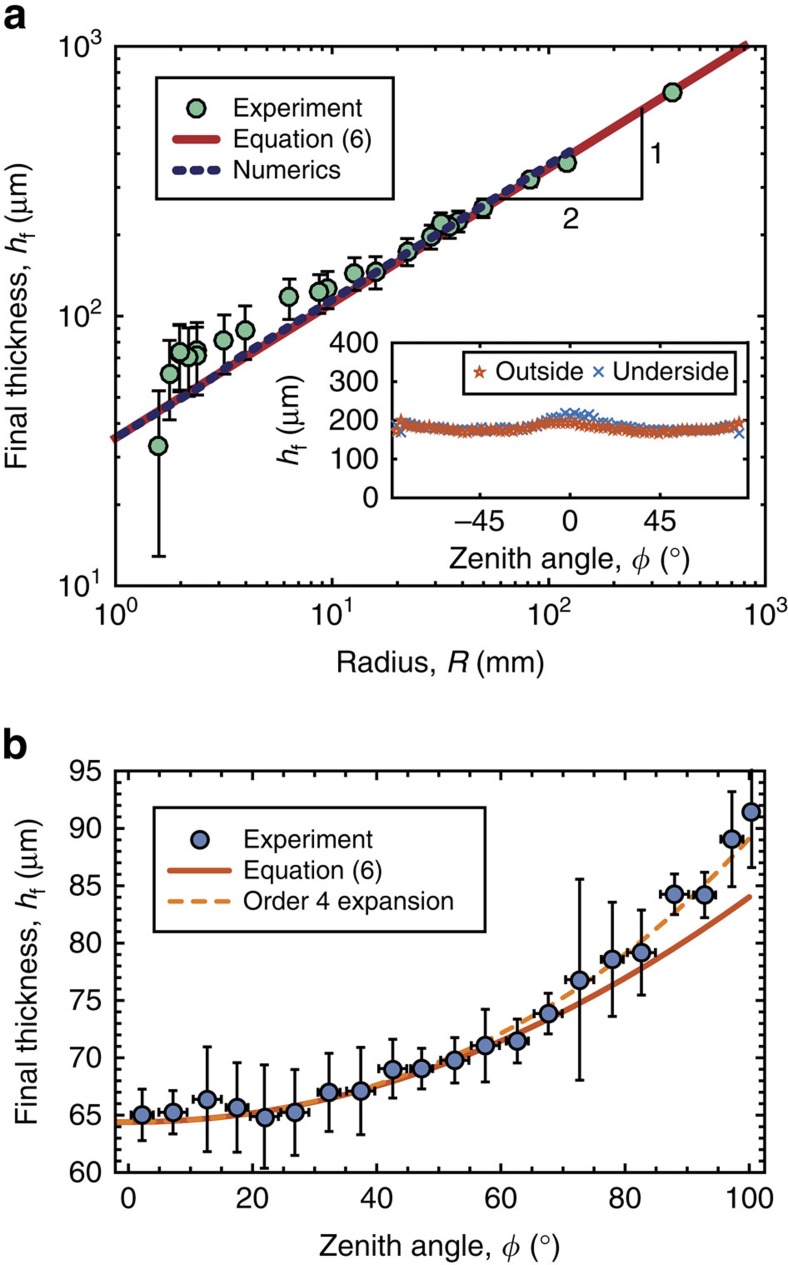
Influence of the geometry on the final shell thickness. (**a**) Comparison between theory, numerics and experiments for the dependence of *h*_f_ on *R*, for the representative case of VPS-32. The results are consistent with the power law *h*_f_∼*R*^1/2^ and in agreement with equations (1) and (6). Inset: Final thickness of shells obtained by pouring VPS-32 on the outside or the underside of a hemisphere with *R*=25 mm. The error bars of the data correspond to the standard deviation of three thickness measurements performed at three different locations of the shell near the apex (*φ*=0°). (**b**) Final thickness of a shell fabricated by pouring PDMS on the outside of a hemisphere with *R*=38 mm compared to equation (6); solid line. The dashed line is the prediction obtained by refining the expansion to the next order, 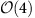, which adds 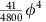 to the terms in the parentheses of equation (6); see Supplementary Note 4 for details. The error bars of the data for *h*_f_ (y-axis) correspond to the standard deviation on multiple measurements. The error bars of the data for *φ* (x-axis) correspond to the size of the angular range used to bin the data.

In Fig. 3a, we compare experimental results (circles) for *h*_f_ of the shells fabricated with VPS-32 to the prediction (solid line) from equation (6) and find good agreement between the two. It is important to note that our model has no adjustable parameters; all numerical coefficients (*α*, *β* and 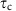) are independently determined once and for all from the viscosity profile and then used in the theory. Note that the profiles obtained when coating either the outside or the underside of complementary spherical molds are nearly identical (Fig. 3a, inset).

In Fig. 3b, we test the shell thickness profile, *h*_f_(*φ*), and find that the experimental results (circles) are in excellent agreement with equation (6).

**Figure 4:**
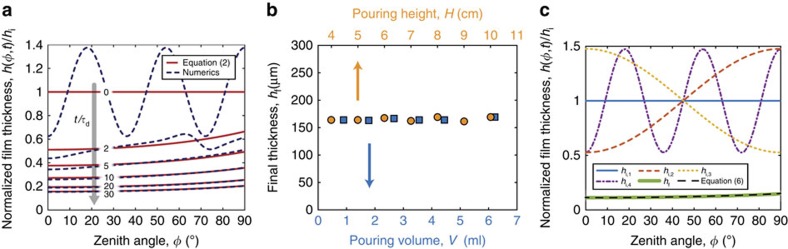
Insensitivity of the coating mechanism to the initial conditions. (**a**) Time-series of the normalized film thickness, *h*(*φ*,*t*)/*h*_i_, with a constant viscosity, at 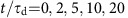, and 30. Dashed lines correspond to the numerically computed evolution of an initially sinusoidal thickness profile of the form *h*(*φ*,*t*=0)/*h*_i_=1+0.375 cos (10*φ*) (see Methods and Supplementary Fig. 7). Solid lines are the theoretical prediction, equation (2), with uniform initial thickness profile, *h*(*φ*,*t*=0)/*h*_i_=1. The various parameters are: average initial film thickness *h*_i_=0.2 mm, sphere radius *R*=20 mm, and material properties for VPS-32. (**b**) Shell thickness, *h*_f_, obtained for different pouring conditions: pouring height, *H*, and volume, *V*, poured onto a spherical mold with *R*=20 mm using VPS-32. (**c**) Four different initial conditions used in the numerics converge to the same final thickness, *h*_f_, and agree well with equation (6) (black dashed line). The simulation parameters are: *h*_i_=2 mm, *R*=38 mm, *h*_i,1_/*h*_i_=1, *h*_i,2_/*h*_i_=1−0.475 cos(2*φ*), *h*_i,3_/*h*_i_=1+0.475 cos(2*φ*), *h*_i,4_/*h*_i_=1−0.475 cos(10*φ*), all with the material properties of VPS-32.

Our theoretical framework is now further validated through numerical simulations (see Methods, as well as Supplementary Figs 6 and 7, for details). The fully nonlinear governing equations (see equation (7) in Methods) were integrated directly, with the appropriate initial conditions and the desired viscosity profile, either constant or time-varying according to equation (5). The results from these numerical simulations are in agreement with both the experimental data and the theoretical predictions for the surface velocity over time and final shell thickness (dashed lines in Fig. 2d and Fig. 3a, respectively). In particular, we have computed the time evolution for a coating film that has an initial sinusoidal thickness profile (dashed line in Fig. 4a), which rapidly converges to the analytically derived equation (2), plotted in Fig. 4a as solid lines for different times.

## Discussion

Our above results establish the basis for the rapid and robust coating process to fabricate spherical elastic shells of nearly uniform thickness, and with radii spanning over two orders of magnitudes (1≤*R*[mm]≤375). As the radius of the sphere is decreased below *R*<10 mm, the agreement between our model and the experiments deteriorates due to the influence of the meniscus that connects the flow on the hemisphere to the puddle that forms as the fluid drains. This effect is not accounted for in our model, but we expect it to be negligible when 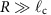, where 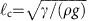 is the capillary length with surface tension, *γ*, that prescribes the relative magnitude of capillary and gravitational forces. Since 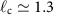 mm and 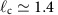 mm for VPS and PDMS, respectively, the deviations of the theory from the data for small *R* are consistent with the onset of these surface tension effects (see Figs 1c and 3a).

**Figure 5:**
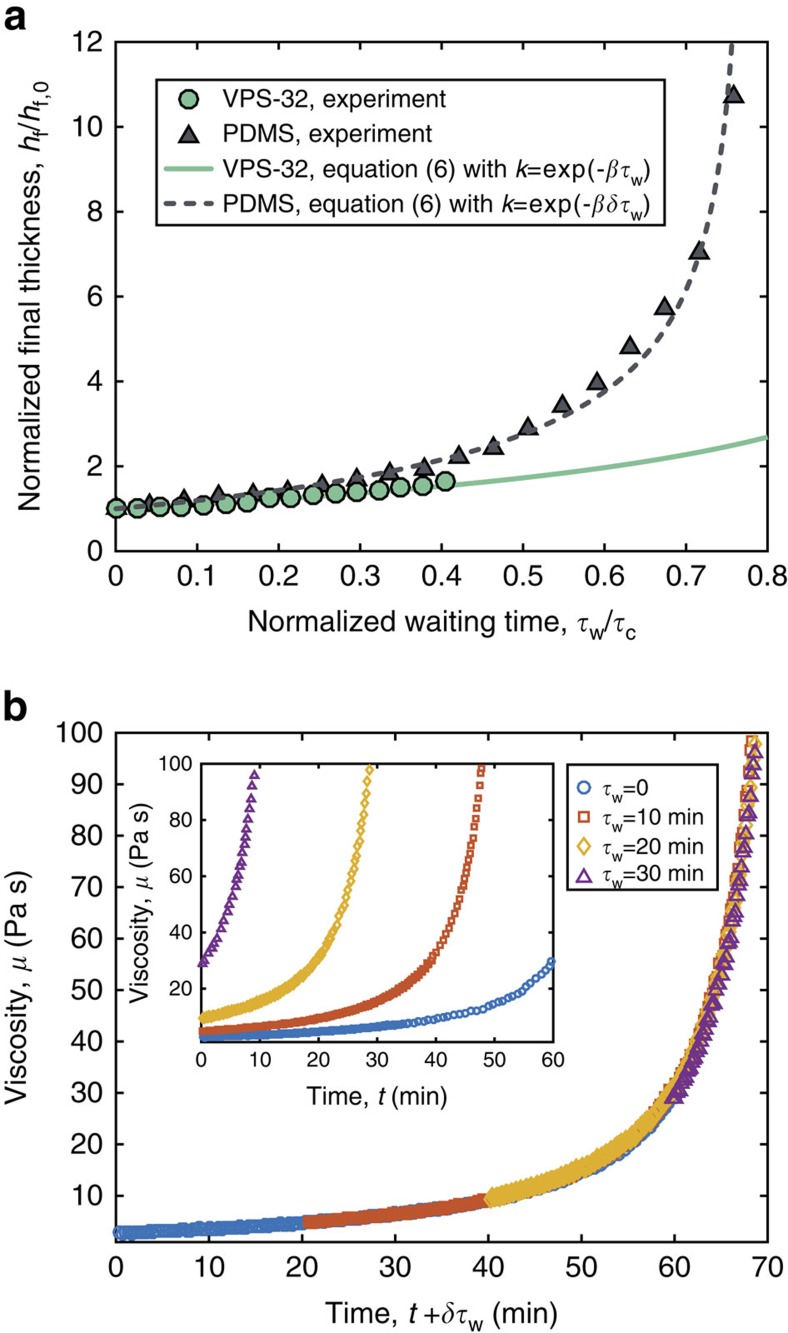
Varying the shell thickness by delaying pouring. (**a**) The shell thickness (normalized by its value *h*_f,0_ when 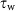
=0) can be tuned by delaying the pouring time by 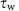 from the moment of preparation of the polymer solution. Results for both VPS-32 and PDMS are shown. (**b**) Viscosity of PDMS versus the sum of the measuring time, *t*, and the effective waiting time, *δ*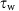 (*δ*=2.02±0.02 from fitting all the curves to the master curve obtained for 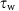
=0). Inset: Viscosity as measured after holding the mixture for a time 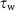 in a quiescent state prior to testing in the rheometer.

Our model, e.g., equation (6), uses the physical parameters for the rheology of the polymer. PDMS was found to behave as a Newtonian fluid for small shear rates (but the viscosity varies with time; see Fig. 5b) whereas VPS exhibited shear thinning (see Supplementary Figs 3 and 4). An estimate of the relevant shear rate is therefore needed. We used the value 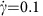 s^−1^, assuming a uniform shear rate across the sample. In reality, 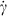 varies from zero at the apex to its peak value at the equator (and also depends on *R*). However, our choice for 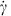 is representative of the applied shear rates over the hemisphere and leads to good agreement between theory and experiments for most values of *R* and *φ*. We have analyzed the sensitivity of the predictions for *h*_f_ with respect to 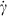 and found that it is small (see Supplementary Note 1).

The variation of the thickness from the pole to the equator of the hemispherical shells was found to be, at most, 6.6% (VPS) and 8.7% (PDMS) from the experiments, 8.7% in the theoretical model, and 8.4% in the numerical analysis (using s.d.). For PDMS, the thickness profile follows equation (6) without any adjustable parameters. This agreement validates our model for the case of time varying viscosities (Fig. 3b). For VPS, shear thinning effects lead to an increase of the thickness at the apex (see Fig. 3a); where the viscosity is largest. These effects are not captured by our model, yet they do not prevent the shell from being uniform within 6.6% variations. We argue that these same effects are the source of the difference between the numerics and the measured free surface velocity for large times (see Fig. 2c).

When the polymer is poured on the underside of a mold, curvature can suppress the Rayleigh-Taylor instability and thereby prevent the formation of dripping droplets^15^. Therefore, the uniformity bounds of the shell that were just stated are ensured, as long as the modified Bond number *B*=*ρgRh*_i_/*γ* (which characterizes the relative importance of gravity and surface tension, *γ*) remains smaller than the critical value, *B*_c_<8 (ref. 15). On the other hand, when the outside of a mold is used, fingering instabilities can occur at the advancing front of the flow, but this can be precluded by pouring a sufficiently large volume of liquid over the surface^14^. A critical volume can be derived^14^ and we estimated it be of the order of 1 ml for an hemisphere of radius *R*=20 mm, in agreement with what was observed experimentally for the sensitivity analysis in Fig. 4b. Under these conditions, pouring on the underside or the outside of complementary molds yields identical shells of the same uniform thickness, *h*_f_. Note that during this process an ≈90% of the volume drains out of the hemisphere. Even if this technique is an excellent rapid-prototyping method, it may not be suitable for large scale industrial applications. Similar limitations are found for spin-coating.

The physical principles that underlay the dynamics of the coating process are rationalized by our analytical model above, to which the separation between the initial drainage and curing timescales is key. Drainage occurs significantly faster than the polymer curing, such that the memory of the flow vanishes before it is arrested by cross-linking to yield the final elastic shell. Consequently, geometry prevails, and the curvature of the mold together with the rheology of the polymer set both the dynamics of the flow and the final thickness of the shell (*h*_f_∼*R*^1/2^). The robustness of this mechanism and its insensitivity to the initial conditions are now corroborated by both experiments and simulations. We measured the thickness of shells obtained for different values of the height from which the polymer is poured onto the mold (4≤*H*[cm]≤10), as well as the volume poured (0.9≤*V*[ml]≤6.3), and find that *h*_f_ is constant to within 5.6% across these various conditions (Fig. 4b). Furthermore, simulations that were initiated with four significantly different initial fluid distributions—uniform, sinusoidal, as well as tapered profiles towards the pole and the equator—all converge to the same final shell thickness, which agrees well with the prediction from equation (6), as shown in Fig. 4c.

Since the final shell thickness is directly connected to the curing time, *h*_f_ can be continuously tuned by waiting a time 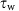 between the preparation of the polymer mixture and the instant when it is poured onto the mold. In Fig. 5a, *h*_f_ is plotted versus 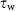, for the representative experiments with both VPS-32 and PDMS. We find that *h*_f_ can be increased by as much as 60% for VPS-32 and elevenfold for PDMS. Substituting 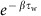 for *k* in equation (6) allows for a direct comparison to the experimental result, with favorable agreement in the case of VPS-32 (solid curve in Fig. 5a). For the PDMS, however, an additional adjustment to our framework is required since we found that its rheology differs if the curing occurred in a quiescent state (e.g., when waiting in bulk for 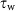 before pouring) versus when sheared (e.g., during coating). Rheometry measurements were performed where the values of 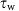 were systematically varied (inset of Fig. 5b). If the time axis for each of these tests is shifted by 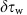 (the constant factor *δ*=2.02±0.02 was determined by fitting), all of the data collapses onto the master curve obtained for 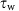
=0. We have therefore concluded empirically that PDMS cures *δ* times faster when quiescent compared to under shear, but we have not been able to find this specific result in the literature. We speculate that the shifting factor required for collapse will likely depend on the shear rate and the specifics of the polymer. With this additional information at hand, substituting 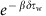 for *k* in equation (6) accounts for the effective waiting time, and yields a prediction for *h*_f_ (dashed line in Fig. 5a) that is in agreement with the experimental data for PDMS. Our model is therefore able to accurately capture the elevenfold continuous variation of the shell thickness obtained when pouring partially cured polymer solutions. As explained in the Supplementary Note 2, we did not have to consider *δ* for VPS-32 because the entire bulk of this more viscous polymer solution is experiencing sustained shear while it was sequentially poured onto a series of identical molds. It is important to note that our theoretical description is only applicable if 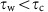.

In summary, we show that coating hemispherical molds with a polymer solution yields thin uniform shells whose thickness can be accurately predicted. Moreover, the final shell thickness can be tuned by modifying the time between polymer preparation and the moment of pouring. The resulting shells are a realization of the drainage dynamics, driven by gravity, slowed down by viscous stresses and eventually arrested by the curing of the polymer. The robustness and flexibility of this mechanism are inherent consequences of the loss of memory in the flow field. The generality of this framework should open the door for future studies to fabricate slender solid structures in a variety of other geometries. A particularly interesting case outside the scope of the current study is the coating of ellipsoidal molds^19^, with two distinct principal curvatures, where the difference between the pouring direction and the orientation of the surface could also play a role. Furthermore, our fabrication technique could be important in the ongoing revival of the mechanics of thin elastic shells, in particularly since it enables fully elastic structures that can reversibly explore strong geometric nonlinearities in their post-buckling regime^20^,^21^,^22^,^23^,^24^,^25^,^26^,^27^,^28^,^29^.

## Methods

### Experiments

Curing of the PDMS (Sylgard 184, Dow Corning) was performed in a convection oven at 20, 35 and 40 °C. The base and curing agent were mixed in a 10:1 weight ratio using a centrifugal mixer for 30 s at 2,000 r.p.m. (clockwise), and then for 30 s at 2,200 r.p.m. (counterclockwise). We sped up the curing process using a cure accelerator (3–6559 Cure Accelerator, Dow Corning) that was mixed to the PDMS elastomer in the weight proportion 5:1 (PDMS-base:Cure-accelerator). VPS (Elite Double 8, 22 and 32, Zhermack, referenced throughout the text as VPS-8, VPS-22 and VPS-32, respectively) was mixed at room temperature (20 °C) with a base/cure ratio 1:1 in weight for 10 s at 2,000 r.p.m. (clockwise), and then 10 s at 2,200 r.p.m. (counterclockwise).

The various polymer solutions (VPS and PDMS) were characterized with a rheometer (AR-G2, TA Instruments) as a function of time and at a constant temperature. The shear rate was fixed at 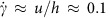 s^−1^, consistently with the characteristic drainage velocity and film thickness (see Supplementary Note 1 and Supplementary Fig. 2). The data for the measured viscosity was then fitted with the piecewise model 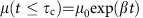 and 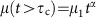 (see Fig. 2d and Supplementary Fig. 1).

The velocity field of the draining polymer was measured using an open-source package for particle imaging velocimetry (PIVlab^30^). A powder spray (Sparkler, Body Shop) was sputtered onto the surface of the flow and imaged using a digital microscope camera (Discovery VMS-004, Veho).

Upon curing, the final thickness, *h*_f_, of the hemispherical elastic shells was measured with an optical microscope after cutting the shell along a meridian using a scalpel (insets of Figs 1c and 3a).

### Model

For the analytical description of the lubrication flow, we consider a hemisphere of radius *R*, initially coated with a fluid of initial average thickness *h*_i_ (*h*(*φ*, 0) may vary spatially). Taking advantage of the azimuthal symmetry, our model is derived in a zenith coordinate system (see schematic in Fig. 2a). By assuming 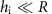 and considering mass conservation, the flow velocity, **u**=(*u*, *v*), can be regarded to be essentially one-dimensional and predominantly tangential to the surface of the sphere in the *φ* direction (the velocity normal to the interface is 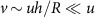). Under low Reynolds number conditions, the lubrication equations^31^ for this flow yield the following nonlinear partial differential equation (see derivation in Supplementary Note 3):


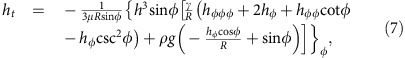


where the subscripts denote differentiation with respect to time and zenith angle, i.e., ∂/∂*t* and ∂/∂*φ*, respectively. Further assuming that the thickness of the fluid film varies slowly along *φ* and that the effects of surface tension are negligible (the latter is valid except close to the moving front), equation (7) can be simplified to^14^


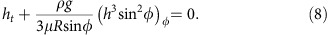


Under the above conditions, the flow is primarily governed by viscous forces and the component of gravity along the flow such that the drainage time, 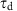, is the relevant time scale of the problem. At the pole (*φ*=0), the thickness varies according to the well established drainage law, 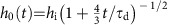 (refs 14, 15), which we generalize further in our problem, in the context of time-varying viscosities.

### Numerical simulations

A numerical procedure was developed to solve equation (7). The zenith angle is discretized uniformly, and we exploit the periodic domain and employ the Fourier spectral method^32^ to compute spatial derivatives with a high degree of accuracy. The effect of numerical diffusion is minimized by performing the time integration with the second-order Crank-Nicolson MATLAB routine ode23t.m. The computational time to derive a solution for a set of geometric and physical parameters is of the order of a few minutes.

To verify the numerics, we compare the numerical solution for an initially uniform film with the analytical solution obtained in the limit 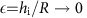 and *φ*=0, namely 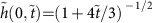, when using *h_i_* and *τ_d_* for nondimensionalization. Supplementary Fig. 6 shows good agreement between the analytical and the numerical solutions, using *N*=256 discretization points.

To complement the comparison between the numerics and the 2nd-order asymptotic solution shown in Fig. 4a, we have obtained results with both methods for sinusoidal initial thickness profiles of the form 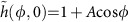 with 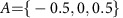. These results are plotted in Supplementary Fig. 7 and confirm that our asymptotic solution is indeed able to predict the correct dynamics for moderate non-uniform film distributions.

## Additional information

**How to cite this article:** Lee, A. *et al*. Fabrication of slender elastic shells by the coating of curved surfaces. *Nat. Commun.* 7:11155 doi: 10.1038/ncomms11155 (2016).

## Supplementary Material

Supplementary Information Supplementary Figures 1-7, Supplementary Table 1, Supplementary Notes 1-5 and Supplementary Reference

Supplementary Movie 1 Representative video of polymer coating process
