# Supplementary material for: Fabrication of slender elastic shells by the coating of curved surfaces
Source: Nat Commun. 2016 Apr 4;7:11155. doi: 10.1038/ncomms11155 (PMC4822016; doi:10.1038/ncomms11155)
Supplement: Supplementary Information — Supplementary Figures 1-7, Supplementary Table 1, Supplementary Notes 1-5 and Supplementary Reference [file ncomms11155-s1.pdf]

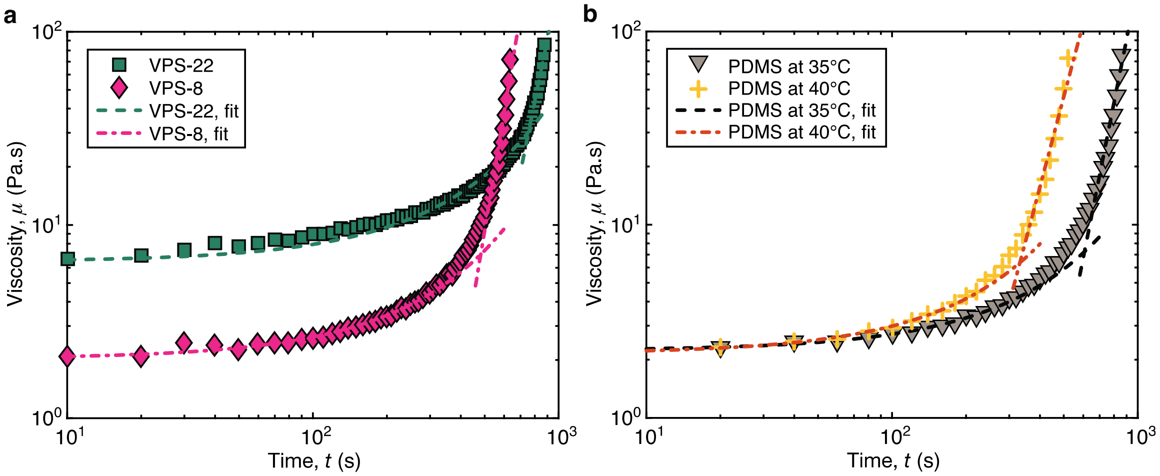

Supplementary Figure 1: Rheology of the polymer solutions. Time dependence of the viscosity for (a) VPS-8 and VPS-22 at 20°C, and (b) PDMS at 35 and 40°C. Data points are experimental data and the dashed lines correspond to fits of twhe theoretical model presented in the main text. The shear rate is fixed at $\dot{\gamma} \approx u/h \approx 0.1\ \mathrm{s}^{-1}$, which is consistent with the flow field during the coating process.

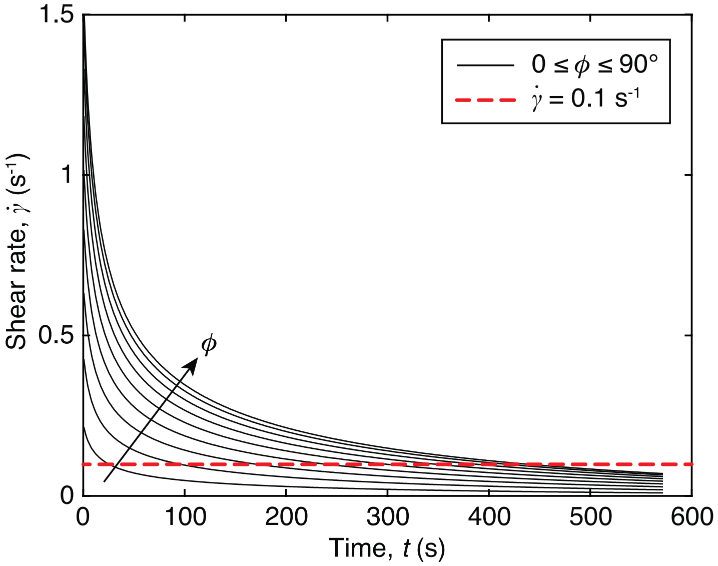

Supplementary Figure 2: Shear rate evolution at different locations. Shear rate evolution estimated as $\dot{\gamma} \approx u(\phi, t)/h(\phi, t)$ for VPS-32 poured on a sphere with R = 38 mm. Within the time period, $\tau_d < t < \tau_c$ (τ_d =6 s is the initial drainage time and τ_d =574 s is the curing time) and over the full range of ϕ, the average value of these various curves is $\dot{\gamma}$= 0.13 s^{-1} and close the value of $\dot{\gamma}$ = 0.13 s^{-1} used in the main text.

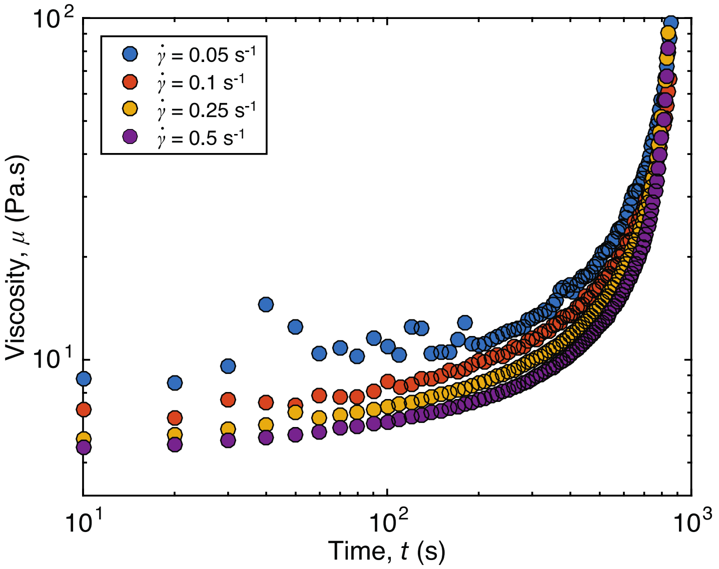

Supplementary Figure 3: Rheology of VPS-32 with different shear rates. Time dependence of the viscosity of VPS-32 with different constant shear rates. The parameters α (±7%), β (±7%), and τ_c (±4%) do not vary much for different shear rates. Only μ_0 varies significantly with different shear rates. μ_0 = {8.6, 7.1, 5.9, 5.6} Pa.s with $\dot{\gamma}$ = {0.05, 0.1, 0.25, 0.5} s $^{-1}$, respectively. The results are indicative of non-Newtonian behavior.

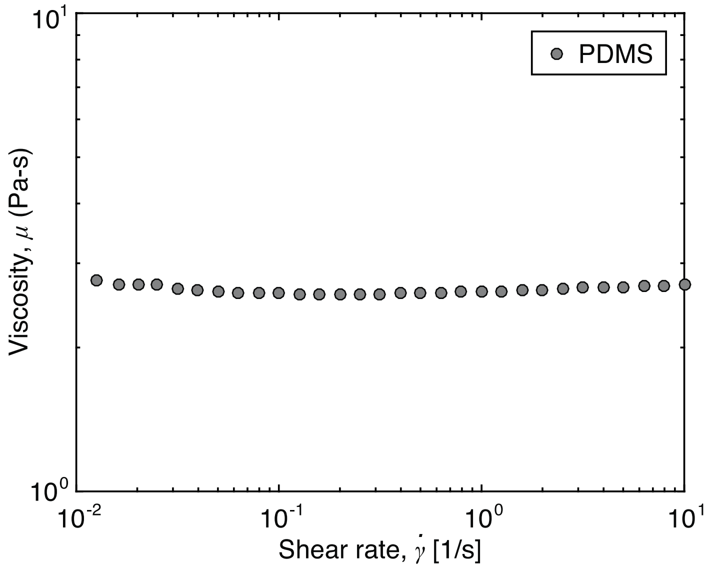

Supplementary Figure 4: Rheology of PDMS with varying shear rate at 20°C. The viscosity of PDMS does not depend on the shear rate when below 10 s^{-1}. All data were measured over a period of 5 min when the effects of curing are still negligible (see Fig. 5b of the main text for the time evolution of μ of PDMS at 20°C).

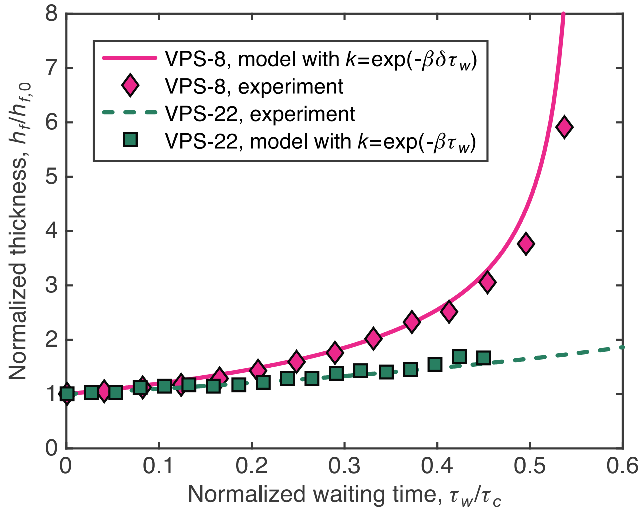

Supplementary Figure 5: Shell thickness can be tuned by the waiting procedure. The final shell thickness (normalized by its value $h_{f,0}$ when $\tau_w = 0$) can be tuned by delaying the pouring time by τ_w from the moment the polymer solution is prepared. The shift factor for VPS-8 is $\delta = 2.1 \pm 0.1$. See Fig. 5a of the main text for the corresponding data for VPS-32 and PDMS.

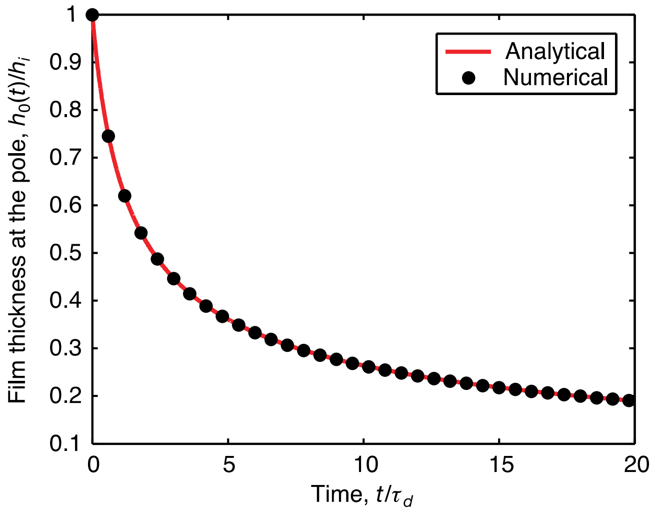

Supplementary Figure 6: Time evolution of the thickness of the film at the pole. Comparison between analytical and numerical solutions for the film thickness evolution at $\phi = 0$ with $N = 256$, $B = 2.3$ and $\varepsilon = 0.01$ (N is the number of discretization nodes, B is the modified Bond number defined in the main text, $\varepsilon = h_i/R$ is the aspect ratio of the film and h_i is the initial average film thickness). These results act as a verification of the simulations with the theoretical model, both of which are validated with experiments in the main text.

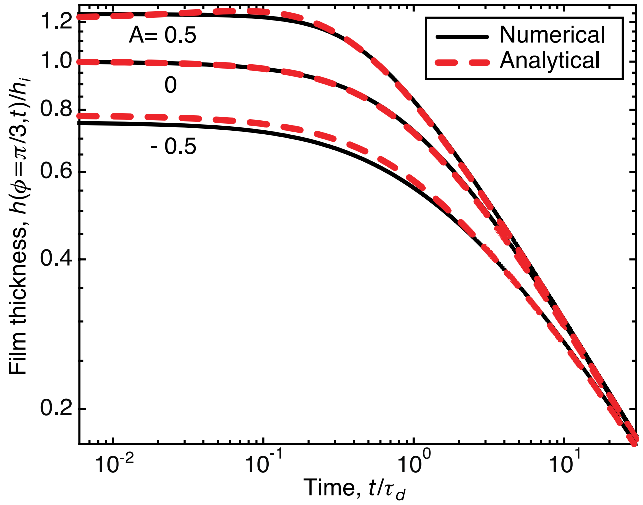

Supplementary Figure 7: Time evolution of the thickness of the film for initially sinusoidal thickness profiles. Comparison between the film thickness evolution computed numerically and the 2nd-order asymptotic solution, both of which for initially sinusoidal thickness profiles, $\tilde{h}(\phi,0) = 1 + A\cos\phi$ with $A = \{-0.5, 0, 0.5\}$. The dimensionless governing parameters for Supplementary Eq. (6) are $\varepsilon = 0.01$ and $B = 2.3$.

Supplementary Table 1: Coefficients for the viscosity model. Values for the initial viscosity μ_0, curing time τ_c, and fitting parameters α and β of our model, for the various polymers used in the experiments (see *Methods* section of the main text for experimental details).

Polymer	μ_0 (Pa.s)	α	β ($\times 10^{-3}$)	τ_c (s)
VPS-32	7.1±0.2	5.3±0.7	2.06±0.09	574±11
VPS-22	6.5±0.3	6.3±0.7	2.05±0.01	738±16
VPS-8	2.0±0.1	7.7±1.2	2.57±0.01	479±7
PDMS, 20°C	2.4±0.1	3.7±0.2	0.560±0.001	2836±81
PDMS, 35°C	2.2±0.1	6.4±1.0	1.94±0.01	613±14
PDMS, 40°C	2.2±0.1	4.8±0.9	3.27±0.03	338±12

Supplementary Note 1: Supporting experimental results on the Polymer Rheology

The initial viscosity, the curing time, and the model fitting parameters for the polymer solutions used in the experiments are presented in Supplementary Table 1. The data for VPS-32 is given in Fig. 2 of the main text for the fixed shear rate of $\dot{\gamma} \approx u/h \approx 0.1$ s^{-1}. The time evolution of the viscosity, as well as the model fitting curves for other liquids are shown in Supplementary Fig. 1.

For the representative case (VPS-32 with R=38 mm) shown in Supplementary Fig. 2, the shear rate $\dot{\gamma} \approx u(\phi, t)/h(\phi, t)$ is a function of ϕ and t and has the average value of 0.13 s^{-1}, for the data within the time period, $\tau_d < t < \tau_c$ (τ_d =6 s is the initial drainage time and τ_d =574 s is the curing time) and over the full range of ϕ. This calculation was conducted using the rheology of VPS measured for $\dot{\gamma}$ =0.1 s^{-1}. Since both of these values (the estimated value and the modeled averaged value) are approximately the same, this choice for the shear rate was referred to as self-consistent in the main text. We measured the viscosity of VPS-32 with different shear rates ranging from 0.05 (close to the limit of the resolution of our rheometer) to 0.5 s^{-1} and the results are shown in Supplementary Fig. 3. The fitting parameters α (±7%), β (±7%), and the curing time τ_c (±4%) do not vary significantly for the explored shear rates. By contrast, the initial viscosity μ_0 does varies in a more pronounced way, as μ_0 = {8.6, 7.1, 5.9, 5.6} Pa.s with $\dot{\gamma}$ = {0.05, 0.1, 0.25, 0.5} s^{-1}, respectively. This is indicative of non-Newtonian behavior. Nevertheless, this changes in the shear rate yields only moderate differences in the final thickness of the shell (owing to square root dependence in equation (6) of the main text). We found the variations in thickness to be of ±10% of the reference case ($\dot{\gamma}$ = 0.1 s^{-1}), even though the shear rate is varied by up to 500% of the reference value. Therefore, the choice of a single shear rate of 0.1 s^{-1} does not compromise our results, and removes an adjustable parameter from the problem.

Unlike VPS that is a shear-thinning liquid, PDMS has a constant viscosity in the range of shear rates that are relevant to the coating process, as shown in Supplementary Fig. 4. Both VPS and PDMS have a time dependent viscosity presented in Fig. 2d and Fig. 5b of the manuscript.

Supplementary Note 2: Discussion on the experimental protocol for delayed pouring

We now provide additional results relevant to the *Discussion* section of the main text regarding the protocol for delayed pouring. In Supplementary Fig. 5 we plot the normalized film thickness, $h_f / h_{f,0}$ (where $h_{f,0}$ is the value of the final thickness when $\tau_w = 0$) as a function of the waiting time, τ_w, normalized by the curing time, τ_c, for VPS-8 and VPS-22.

For convenience, we reproduce equation (6) from the main text, for the final thickness, as a function of the various physical and geometric parameters

$$h_f = \sqrt{\frac{3\mu_0 R}{4\rho g}\frac{1}{K}}\left(1+\frac{\phi^2}{10}\right), \tag{1}$$

with $K = \{(k - \mathrm{e}^{-\beta\tau_c})/\beta\} + \{\tau_c e^{-\beta\tau_c}/(\alpha - 1)\}$, and $k = e^{-\beta\tau_w}$ (or k=1) with (or without) the waiting procedure between the preparation and coating of polymer solution, respectively. For more details about its derivation, see Supplementary Note 5, below.

For low viscosity polymer solutions (PDMS and VPS-8) the shift factor δ is needed to predict the final thickness. We speculate that, when waiting in bulk in a container, the boundary layers are thin compared with the size of the container due to the low viscosity of these polymers. Consequently, the majority of the polymer solution is not disturbed by the movement of the container, which is therefore in a quiescent state until τ_w. On the other hand, the higher viscosity of VPS-32 and 22, results in the diffusion of shear in the entire bulk of the solution during the waiting time, τ_w, while the bulk of the polymer solution is sequentially and continuously poured onto a series of identical molds. Thus, the theoretical model without a shift factor agrees well with the experimental results of VPS-32 and 22, but δ is finite for the other polymers (see *Discussion* in the main text).

Supplementary Note 3: Supporting analytical results for the lubrication model

The derivation of the underlying equation of our model presented in the *Methods* of the manuscript is briefly outlined in this section. We assume a thin liquid film on a sphere of radius, R, invariant in the azimuthal direction. Its initial characteristic thickness of the film is h_i; the resulting film aspect ratio is $\varepsilon = h_i/R$. As mentioned in the *Discussion* section of the main text, the time evolution of a thin-film on the outside and underside of the mold produces identical results. Here, we focus on the derivation of the first case. Considering a small aspect ratio ε of the film, mass conservation indicates that the velocity normal to the interface is significantly smaller than the tangential component. Furthermore, the low Reynolds number conditions for this flow allows for the Stokes equations to be used. The equation for momentum balance in the radial direction is

$$0 = -\frac{1}{\rho}\frac{\partial p}{\partial r} - g\cos\phi, \tag{2}$$

and the boundary condition for the pressure is $p(R+h) = p_0 + \gamma\kappa$ (p_0 is the external pressure, γ is the surface tension of the fluid, and κ is the curvature of the interface). Integrating Supplementary Eq. (2) along the radial direction and using the above boundary condition yields the pressure distribution, $p(r, \phi) = p_0 + \gamma\kappa + \rho g \cos\phi\,(R + h - r)$. By integrating twice, the ϕ component of the momentum equation,

$$0 = -\frac{1}{\rho r}\frac{\partial p}{\partial \phi} + \nu\frac{1}{r^2}\frac{\partial}{\partial r}\left(r^2\frac{\partial u}{\partial r}\right) + g\sin\phi, \tag{3}$$

and considering the no-slip boundary condition at the sphere surface, $u(R, \phi) = 0$, as well as the zero-shear stress interface, $\partial u(R + h, \phi)/\partial r = 0$, we obtain the tangential velocity component:

$$u(r,\phi) = \left(-\frac{\gamma\kappa_\phi}{\mu R} - \frac{\rho g\cos\phi h_\phi}{\mu R} + \frac{\rho g\sin\phi}{\mu}\right)\left(h - \frac{r-R}{2}\right)(r-R). \tag{4}$$

The depth-integrated velocity is given by $Q(\phi) = \int_R^{R+h} u(r,\phi)dr$. Using the local mass conservation in spherical coordinates, $\partial h/\partial t + (R\sin\phi)^{-1}\partial(\sin\phi\, Q)/\partial\phi = 0$, we eventually obtain the lubrication equation:

$$h_t + \frac{1}{3\sin\phi\,\mu R}\left[h^3\sin\phi\left(-\underbrace{\frac{\gamma\kappa_\phi}{R}}_{\mathrm{I}} - \underbrace{\frac{\rho g\cos\phi\, h_\phi}{R}}_{\mathrm{II}} + \underbrace{\rho g\sin\phi}_{\mathrm{III}}\right)\right]_\phi = 0, \tag{5}$$

where the leading order curvature derivative is $\kappa_\phi = -R^{-2}(h_{\phi\phi\phi} + 2h_\phi + h_{\phi\phi}\cot\phi - h_\phi \csc^2\phi)$. The term **I** in the spatial variation of the flux corresponds to the surface tension effects, term **II** represents the variation of the hydrostatic pressure distribution and term **III** accounts for the drainage. In the case of a liquid film on the underside of a sphere, the hydrostatic pressure variation term would have an opposite sign, but the rationale would otherwise be identical.

The film thickness and time can be non-dimensionalized by h_i and the initial drainage time, $\tau_d = \mu R/(\rho g h_i^2)$, respectively, such that the lubrication equation expressed with non-dimensional quantities is written as

$$\tilde{h}_{\tilde{t}} + \frac{1}{3\sin\phi}\left\{\tilde{h}^3 \sin\phi\left[\frac{\epsilon^2}{B}\left(\tilde{h}_{\phi\phi\phi} + 2\tilde{h}_\phi + \tilde{h}_{\phi\phi}\cot\phi - \tilde{h}_\phi \csc^2\phi\right) - \epsilon\tilde{h}_\phi\cos\phi + \sin\phi\right]\right\}_\phi = 0, \quad (6)$$

where $B = \rho g h_i R/\gamma$ is the modified Bond number.

For a time-varying viscosity, the initial drainage time is built upon μ_0 and the factor $\mu_0/\mu(t)$ appears ahead of the flux variation terms.

From the mean velocity, the velocity at the interface can be computed as:

$$u(\phi,t) = \frac{3}{2}\langle u(\phi,t)\rangle = \frac{3}{2}\frac{Q(\phi,t)}{h(\phi,t)}. \quad (7)$$

Supplementary Note 4: Derivation of the asymptotic nonlinear drainage flow solution

The nonlinear drainage flow solution presented in the *Results* section of the main text is derived next. Assuming that the depth of the fluid varies slowly along the substrate and that the effect of surface tension is negligible, which are both valid assumptions except close to the moving front at short times, we obtain the simplified version of the Supplementary Eq. (6) [1]:

$$\tilde{h}_{\tilde{t}} + \frac{1}{3\sin\phi}\left(\tilde{h}^3 \sin^2\phi\right)_\phi = 0. \tag{8}$$

This equation confirms that, under the aforementioned assumptions, the dynamics of the thin-film does not depend on whether it is formed on the underside or outside of the substrate. The velocity field is given by $\tilde{u}(\phi,\tilde{t}) = \tilde{h}(\phi,\tilde{t})^2 \sin\phi\,/2\epsilon$ (the tilde represents dimensionless quantities), or in dimensional form:

$$u(\phi,t) = \frac{\rho g h(\phi,t)^2}{2\mu}\sin\phi. \tag{9}$$

Furthermore, we postulate an expansion solution of the form $\tilde{h}(\phi,\tilde{t}) = \tilde{h}_0(\tilde{t}) + \phi^2\tilde{h}_2(\tilde{t}) + \phi^4\tilde{h}_4(\tilde{t}) + O(\phi^6)$, where odd terms vanish due to symmetry reasons. The zeroth-order solution, $\tilde{h}_0(\tilde{t}) = (1+4\tilde{t}/3\,)^{-1/2}$, is well known (Refs. [24,25] of the main text) and corresponds to the exact solution of Supplementary Eq. (8) at the pole (*i.e.*, $\phi = 0$). By substituting this expansion into Supplementary Eq. (8) and further developing $\cos\phi \sim 1 - \phi^2/2 + \phi^4/24 + O(\phi^6)$ and $\sin\phi \sim \phi - \phi^3/6 + O(\phi^5\,)$, we obtain a differential equation for each order. The equation for $\tilde{h}_2$ is:

$$\frac{d\tilde{h}_2}{d\tilde{t}} + 4\tilde{h}_0^2\tilde{h}_2 = \frac{1}{3}\tilde{h}_0^3. \tag{10}$$

The particular solution is assumed to be of the form $\tilde{h}_2^P(\tilde{t}) = a(1+4\tilde{t}/3)^{-1/2}$. Substituting this form into Supplementary Eq. (10) results in an equation for the parameter *a*, yielding *a* = 1/10. On the other hand, the homogeneous solution of the equation is $\tilde{h}_2^H(\tilde{t}) = b(1+4\tilde{t}/3)^{-3}$. The second order solution of Supplementary Eq. (8), $\tilde{h}(\phi,\tilde{t}) \approx \tilde{h}_0(\tilde{t}) + \phi^2\left[\tilde{h}_2^P(\tilde{t}) + \tilde{h}_2^H(\tilde{t})\right]$, can be eventually shown to read

$$\tilde{h}(\phi,\tilde{t}) \approx \frac{1}{\sqrt{1+\frac{4}{3}\tilde{t}}}\left[1 + \frac{\phi^2}{10}\left(1 + c\left(1+\frac{4}{3}\tilde{t}\right)^{-5/2}\right)\right], \tag{11}$$

where the parameter c depends on the initial condition.

Note that the homogeneous solution only influences the transient regime; for large times the solution decreases as $(4\tilde{t}/3)^{-1/2}$ and is independent of the initial condition. In dimensional form, the asymptotic solution for the film thickness is given by:

$$h(\phi,t) \approx \frac{h_i}{\sqrt{1+\frac{4}{3}\frac{t}{\tau_d}}}\left[1+\frac{\phi^2}{10}\left(1+c\left(1+\frac{4}{3}\frac{t}{\tau_d}\right)^{-5/2}\right)\right]. \tag{12}$$

A more accurate solution is obtained when considering the fourth-order term in the expansion. The equation for $\tilde{h}_4$ is:

$$\frac{d\tilde{h}_4}{d\tilde{t}} + 6\tilde{h}_0^2\,\tilde{h}_4 = \frac{4}{3}\tilde{h}_0^2\,\tilde{h}_2 - 6\,\tilde{h}_0\tilde{h}_2^2 - \frac{1}{36}\tilde{h}_0^3. \tag{13}$$

Proceeding in a similar way as for the second-order problem, the fourth-order solution reads:

$$\tilde{h}_4(\tilde{t}) = \frac{41/4800}{\sqrt{1+\frac{4}{3}\tilde{t}}}\left[1+\frac{216c^2}{41}\left(1+\frac{4}{3}\tilde{t}\right)^{-5} + \frac{32c}{41}\left(1+\frac{4}{3}\tilde{t}\right)^{-5/2} + d\left(1+\frac{4}{3}\tilde{t}\right)^{-4}\right], \tag{14}$$

where the parameter d depends on the initial condition. At late times, the spatial variation of the film thickness is therefore of the form $1+(1/10)\phi^2+(41/4800)\phi^4+\mathcal{O}(\phi^6)$.

Supplementary Note 5: Considering curing effects for the predictions of the final film thickness

In order to obtain the prediction for the final film thickness given by equation (6) in the main text, Supplementary Eq. (12) needs to be modified to take into account the rheology of the polymer fluid. For this purpose, we make use of the following empirical description for the evolution of the viscosity, which we found to fit our experimental data well:

$$\mu(t) = \begin{cases} \mu_0 \exp(\beta t), & \text{if } t \le \tau_c, \\ \mu_1 t^{\alpha}, & \text{if } t > \tau_c, \end{cases} \tag{15}$$

with $\mu_1 = \mu_0.\exp(\beta\tau_c)\tau_c^{-\alpha}$, where τ_c is the curing time and α and β are parameters which have to be fitted depending on the specific details of the fluid (see Supplementary Table 1 for the numerical values for the polymers used in our experiments). Note that because of the small value of the parameter β, the viscosity can be initially assumed as constant (see the *Results* section of the main text). The asymptotic estimates for the film thickness and for the film surface velocity resulting from this viscosity model and Supplementary Eqs. (12) and (9) are now derived.

During the curing regime, $\tau_c << t$, the film thickness is written as

$$h(\phi, t) \approx \frac{h_i}{\sqrt{1 + \frac{4}{3}\frac{\rho g h_i^2}{R}\int_0^t \frac{1}{\mu(t')}dt'}}\left(1 + \frac{\phi^2}{10}\right), \tag{16}$$

or

$$h(\phi, t) \approx \frac{h_i\left(1 + \frac{\phi^2}{10}\right)}{\sqrt{1 + \underbrace{\frac{4}{3}\frac{\rho g h_i^2}{R}\frac{(\alpha - 1)(e^{\beta\tau_c} - 1) + \tau_c\beta}{\beta\mu_0 e^{\beta\tau_c}(\alpha - 1)}}_{\mathbf{I}} - \underbrace{\frac{4}{3}\frac{\rho g h_i^2}{R}\frac{\tau_c^{\alpha}}{\mu_0 e^{\beta\tau_c}(\alpha - 1)}t^{1-\alpha}}_{\mathbf{II}}}}. \tag{17}$$

If the curing time τ_c is large enough so that the term **I** is much larger than unity and if the final time t is larger than τ_c so that term **II** becomes negligible, then the asymptotic solution for the film thickness is given by

$$h_f(\phi) \approx \sqrt{\frac{3\mu_0 R}{4\rho g}\frac{\beta e^{\beta\tau_c}(\alpha - 1)}{(\alpha - 1)(e^{\beta\tau_c} - 1) + \tau_c\beta}}\left(1 + \frac{\phi^2}{10}\right) = \sqrt{\frac{3\mu_0 R}{4\rho g}\frac{1}{K}}\left(1 + \frac{\phi^2}{10}\right), \tag{18}$$

with $K = \left(k - e^{-\beta\tau_c}\right)/\beta + \tau_c e^{-\beta\tau_c}/(\alpha - 1)$, which is identical to equation (6) in the main text. The tuned final thickness by delayed pouring can be obtained by changing the bounds of the integral $\int_0^t \frac{1}{\mu(t')} dt'$ to $\int_{\tau_w}^t \frac{1}{\mu(t')} dt'$ in Supplementary Eq. (16), which results in $k = e^{-\beta\tau_w}$. The velocity estimate then becomes

$$u(\phi, t) \approx \frac{3}{8} \frac{\beta(\alpha - 1)\tau_c^{\alpha}}{(\alpha - 1)(e^{\beta\tau_c} - 1) + \tau_c\beta} \frac{R}{t^{\alpha}} \left(1 + \frac{\phi^2}{10}\right)^2 \sin\phi$$

$$= \frac{3}{8} \frac{\tau_c^{\alpha}}{e^{\beta\tau_c} K} \frac{R}{t^{\alpha}} \left(1 + \frac{\phi^2}{10}\right)^2, \qquad (19)$$

where we highlight the fast temporal decrease of the velocity $u \sim t^{-\alpha}$ and its independence on the viscosity, density and gravity.

Supplementary Reference:

[1] Takagi, D. and Huppert, H. E. Flow and instability of thin films on a cylinder and sphere. *J. Fluid Mech.* **647,** 221-238 (2010).
